# Synthesis of *Escherichia coli* OmpA Oral Nanoparticles and Evaluation of Immune Functions against the Major Etiologic Agent of Cow Mastitis

**DOI:** 10.3390/vaccines9030304

**Published:** 2021-03-23

**Authors:** Xiang Liu, Wei Sun, Nana Wu, Na Rong, Chao Kang, Sijie Jian, Chunlin Chen, Chen Chen, Xiaoying Zhang

**Affiliations:** 1 College of Veterinary Medicine, Northwest A&F University, Yangling 712100, China; liuxiang888525@163.com; 2 Chinese-German Joint Institute for Natural Product Research, College of Biological Science and Engineering, Shaanxi University of Technology, Hanzhong 723000, China; sww17809267113@163.com (W.S.); wunana0413@163.com (N.W.); rongna0628@163.com (N.R.); m18142347756@163.com (C.K.); jianjiesi123@gmail.com (S.J.); chen2362505579@163.com (C.C.)

**Keywords:** *E. coli*, cow mastitis, OmpA protein nanoparticles, immune function, oral vaccine

## Abstract

*Escherichia coli* is a major etiologic agent of cow mastitis, a condition that results in huge economic losses. There is a lack of an oral vaccine for cow mastitis. Previous studies have confirmed that the outer membrane protein A (OmpA) of *E. coli* is immunogenic and can be used for vaccine design. In the present study, OmpA was encapsulated into nanoparticles (NP-OmpA) for an oral vaccine for cow mastitis. **Methods:** OmpA was purified with Ni-NTA flow resin and encapsulated with chitosan (CS) to prepare NP-OmpA nanoparticles. The gastrointestinal tract was simulated in vitro (PBS, pH 1.2) to measure the protein release rate. The optimal preparation conditions for NP-OmpA were determined by analyzing the concentrations of OmpA and CS, magnetic mixing speed, mixing time, and the ratio of tripolyphosphate (TPP)/CS (*w/w*). NP-OmpA safety was assessed by function factors and histopathological examination of livers and kidneys. The immune activity of NP-OmpA was determined using qRT-PCR to assess immune-related gene expression, leukocyte phagocytosis of *Staphylococcus aureus*, ELISA to evaluate antiserum titer and immune recognition of *E. coli*, and the organ index. The immune protection function of NP-OmpA was assessed by the protection rate of NP-OmpA to *E. coli* in mice, qRT-PCR for inflammation-related gene expression, assay kits for antioxidant factors, and visceral injury in the histopathological sections. **Results:** NP-OmpA nanoparticles had a diameter of about 700 nm, loading efficiency (LE) of 79.27%, and loading capacity (LC) of 20.31%. The release rate of NP-OmpA (0~96 h) was less than 50% in vitro. The optimal preparation conditions for NP-OmpAs were OmpA protein concentration of 2 mg/mL, CS concentration of 5 mg/mL, TPP/CS (*w/w*) of 1:1, magnetic mixing speed of 150 r/min, and mixing time of 15 min. Histopathological sections and clinical analytes of uric acid (UA), creatinine (Cr), alanine aminotransferase (ALT), aspartate transaminase (AST), catalase (CAT), glutathione (GSH), and malondialdehyde (MDA) showed NP-OmpA did not damage mice livers or kidneys. NP-OmpA could enhance the immune-related gene expression of IFN-γ and HSP70 in the spleen, liver, and kidney and the leukocyte phagocytosis of *S. aureus*. The antiserum titer (1:3200) was obtained from mice immunized with NP-OmpA, which had an immune recognition effect to *E. coli*. The immune protection rate of NP-OmpA was 71.43% (*p* < 0.05) to *E. coli*. NP-OmpA could down-regulate the inflammation-related gene expression of TNF-a, IL-6, and IL-10 in the spleen, liver, and kidney, and the antioxidant factors MDA and SOD in the liver, and reduce injury in the liver and kidney of mice induced by *E. coli*. **Conclusions:** A novel NP-OmpA nanoparticle was encapsulated, and the optimal preparation conditions were determined. The NP-OmpA was safe and had good immune functions. They are expected to induce a response that resists infection with the major etiologic agent (*E. coli*) of cow mastitis.

## 1. Introduction

*Escherichia coli* is a gram-negative bacterium that widely exists in the natural environment and can enter the animal body through the skin or digestive tract [1]. *E. coli*, together with *Staphylococcus aureus* and *Streptococcus*, are the major etiologic agents that cause dairy cow mastitis, which results in huge economic losses in the dairy industry [2,3]. It can also induce diseases such as septicemia, pericarditis, aerocyst, ophthalmia, and omphalitis in chickens [4,5] and causes hemolytic uremia, neonatal septicemia, and meningitis in humans [6,7,8]. Thus, *E. coli* is an opportunistic zoonotic pathogen. At present, antibiotics are the most common drugs used to prevent and treat *E. coli* infection. However, the abuse of antibiotics will inevitably lead to bacterial resistance, drug residues, and environmental pollution and will also affect the microecological balance of animal intestinal flora [9,10]. Therefore it is necessary to develop new drugs to prevent and treat *E. coli* infection.

Outer membrane protein A (OmpA) is the main outer membrane protein (OMP) of gram-negative bacteria. It consists of an N-terminal transmembrane domain (1–171) and a C-terminal cytoplasmic domain (172–325) and is genetically highly conserved. OmpA plays an important role in biofilm formation, host cell invasion, pore formation, and multidrug resistance [11]. More specifically, *E. coli* OmpA plays a key role in pathogenicity and is the main virulence factor in *E. coli* infection [12]. OmpA also has strong immunogenicity and can induce innate and adaptive immune responses in animal hosts. OmpA can regulate the expression of cytokines, chemokines, nitric oxide synthase, and cyclooxygenase-2 and protect mice from death caused by *E. coli* infection [13]. Anti-OmpA antibodies can regulate the function of specific phagocytosis to protect against *E. coli* infection [14]. We found that OmpA had significant protective rates of 58.33% and 46.15% against *E. coli* and *Staphylococcus aureus,* respectively, and the OmpA fragment is also immunogenic [15]. Therefore, OmpA is a vaccine candidate for the prevention of *E. coli* infection.

To further improve the immune function of OmpA and to produce a formulation that could survive degradation in the gastrointestinal tract with sustained release and enhanced efficacy, we applied a nano preparation method to encapsulate OmpA with chitosan (CS) to encapsulate nanoparticles.

## 2. Materials and Methods

### 2.1. Animals and Bacterial Strains

Kunming mice (4 weeks old) were purchased from Chongqing Tengxin Biotechnology Co. Ltd., China. All animal procedures were performed in accordance with the guidelines prescribed in the Guide for the Care and Use of Laboratory Animals and were approved by the Institutional Animal Ethics Committee, Shaanxi University of Technology, China (No. 2019-015).

*E. coli* and *S. aureus* isolated from cow mastitis and the *E. coli* OmpA expression strain were all preserved in the biochemistry and molecular laboratory of the Shaanxi University of Technology.

### 2.2. Expression, Purification, and Preparation of Nanoparticles of OmpA

Expression and purification of OmpA were performed as described previously [16]. Briefly, the OmpA expression strain was cultured overnight and transferred to 600 mL LB medium until OD_600_ nm = 0.5. Isopropyl-β-d-thiogalactoside (IPTG) was then added and induced at 20 ℃ for 24 h. Bacterial cells were harvested by centrifugation and disrupted by sonication with an ice bath. Finally, OmpA was purified with the Ni-NTA flow resin (Sigma, St. Louis, MO, USA).

The OmpA nanoparticles (NP-OmpA) were prepared by CS encapsulation. Briefly, tripolyphosphate (TPP) (3 mL, 1 mg/mL) was added dropwise to a CS solution (10 mL, 1 mg/mL), and stirred for 10 min at 700 r/min. After centrifugation (15 min at 9500 r/min), the precipitate was added to 25 mL of water and subjected to ultrasound (2 min at 50% power). Then 3 mL of OmpA was added dropwise. After centrifugation, 10 mL of water was added to the precipitate to obtain the NP-OmpA. Nanoparticle diameter and zeta potential were analyzed using a Laser Particle Size Analyzer (Beckman, Fullerton, CA, USA), and the morphology was observed using a scanning electron microscope (Phenom Pro, Eindhoven, The Netherlands) [17].

### 2.3. In Vitro Release of NP-OmpA

NP-OmpA was analyzed for in vitro protein release to simulate the digestive function of the gastrointestinal tract. Briefly, the NP-OmpA solution was transferred to a dialysis bag (MW 14–20 kDa) that was placed into a pH 1.2 solution. At each assigned time point (0, 2, 4, 6, 8, 10, 12, 14, 16, 18, 20, 22, 24, 36, 48, 60, 72, 84, and 96 h), 200 μL of supernatant was taken from the solution and analyzed for protein content using Bradford diagnostic kits [18].

### 2.4. The Optimal Preparation Conditions for NP-OmpA

Nanoparticles were prepared as described by Li et al. [17], with minor modifications. The parameters that were optimized for the preparation of the NP-OmpA were the concentrations of OmpA and CS, magnetic mixing speed, mixing time, and the ratio of TPP/CS (*w/w*). In brief, (1) The concentration of OmpA: TPP (3 mL, 0.5 mg/mL) was added dropwise into a CS solution (10 mL, 0.5 mg/mL) with stirring for 10 min at 700 r/min. After centrifugation (15 min at 9500× *g*), 25 mL of water was added to the precipitate under continuous ultrasonication for 2 min, and 3 mL OmpA solution (0.5, 1.0, 1.5, 2.0, 2.5 mg/mL) was added dropwise with stirring (150 r/min for 15 min). After centrifugation (15 min at 9500 r/min), nanoparticles were obtained. (2) The mixture of CS:TPP was added into the CS solution (1.0, 2.0, 3.0, 4.0 and 5.0 mg/mL), and mixed for 10 min at 700 r/min. After centrifugation, 25 mL of water was added to the precipitate under continuous ultrasonication, and 3 mL of OmpA solution was added. Finally, nanoparticles were obtained by centrifugation. (3) The ratio of TPP/CS:TPP was added into the CS solution at the ratios of TPP/CS (*w/w*) of 1:1, 1:2, 1:3, 1:4, and 1:5. After centrifugation, the precipitate was resuspended, and the OmpA solution was added. Finally, nanoparticles were obtained by centrifugation. (4) Magnetic mixture speed: After centrifugation, the precipitate was resuspended with dH_2_O, and the OmpA solution was added dropwise with magnetic mixing speeds of 100, 150, 300, 500, and 700 r/min. Finally, nanoparticles were obtained by centrifugation. (5) Mixing time: After centrifugation, the precipitate was resuspended, and then OmpA solution was added with mixing times of 10, 15, 20, 30, 60, and 120 min. Finally, nanoparticles were obtained by centrifugation. Loading efficiency (LE), loading capacity (LC), and particle diameters were also measured to determine the optimal parameters for the preparation of NP-OmpA.

### 2.5. Immunoprotective Effect of NP-OmpA

Kunming mice were divided into four groups of 20 in each. Groups 1-4 were vaccinated with NP-OmpA, OmpA, CS nanoparticles without OmpA (NP-Empty), and normal saline (NC), respectively. After pilot experimental exploration and optimization, all vaccines were orally administered at 6 µg/g of mice body weight, four times. The first immune interval was 14 days, and the subsequent immune interval was 7 days. Mice were intraperitoneally challenged with *E. coli* at 1.0 × 10^8^ CFU at day 7, after the fourth immunization, and the mouse mortality was counted for 15 days post *E. coli* challenge, and the immune protection rate (RPS) of the mice was calculated, RPS (%) = [1 − (% vaccinated mortality/% control mortality)] × 100. SPSS software was used for statistical analysis [19]. In addition, in each group, 15 mice were used for the immune protection analysis, and 5 were used for the assessment of inflammation-related genes by qRT-PCR, WBC counting, organ index, leukocyte phagocytosis, antibody titer, and histopathology observation.

### 2.6. Organ Index, White Blood Cell (WBC) Count, and Leukocyte Phagocytosis

The organ index was implemented as follows: the mice were weighed after cervical dislocation. The spleen and thymus were removed and weighed. The organ index = (organ weight/mice weight) × 100 (mg/g).

WBC counts were conducted as follows: mice anticoagulant was collected to prepare blood smears. Wright’s and Giemsa’s dye solutions were used for staining, and samples were washed slowly for 3 min with water. After drying, microscopic counting was performed.

Leukocyte phagocytosis was performed as described previously [20]. Briefly, 0.2 mL of mice anticoagulant was added to 2 mL of *S. aureus* (6 × 10^8^ CFU/mL) and shaken for 60 min at 25 ℃ in a water bath. The mixed liquid smears were drawn with a pipette. Each sample was fixed with methanol for 3–5 min, stained (Giemsa) for 30 min, washed and air dried, and then observed by oil microscope. Phagocytic percentage (*PP* %) = no. of WBCs involved in phagocytosis per 100 leukocytes/100 × 100%. Phagocytic index (*PI* %) = no. of bacteria phagocytized/no. of WBCs phagocytizing bacteria. The results were analyzed by variance analysis (ANOVA) and the Tukey test (*p* < 0.05) with SPSS 19.0 software.

### 2.7. Detection of the Interaction between Antiserum and E. coli, and Antiserum Titer

NP-OmpA antiserum was harvested on day 7 after the fourth post-vaccination. Interaction between the antiserum and bacteria was assessed by ELISA as described previously [19]. Briefly, after *E. coli* were harvested, 1% formaldehyde (*w/v*) was added for 90 min at 80 °C to inactivate the bacteria, and the solution was adjusted until *OD*_600_ nm = 0.2. The bacterial solution was transferred to 1.5 mL tubes, and antisera at various dilutions were added before incubation for 1 h at 37 °C. After washing with PBS, rabbit anti-mouse antibody (Sigma, St. Louis, MO, USA) was added, and the solution was washed with PBS again. The bacteria were suspended with 20 μL of PBS and transferred to an enzyme-linked plate. Coloration liquid (50 µL H_2_O_2_ and 50 µL TMB) and stop solution (50 μL 2M H_2_SO_4_) was added to the wells, and absorbance was read at *OD*_450_ nm with a microplate reader (Bio-Rad, Hercules, CA, USA).

Serum antibody titer was evaluated by ELSA as described previously [19]. Briefly, the purified OmpA was added to an enzyme-linked plate and incubated with blocking solution (5% skim milk), and various dilutions of antiserum were added before incubation for 1 h at 37 °C. After washing, rabbit anti-mouse antibody (Sigma, St. Louis, MO, USA) was added to the plate. Coloration liquid (50 µL H_2_O_2_ and 50 µL TMB) was added to each well, and the absorbance read at *OD*_450_ nm with a microplate reader (Bio-Rad, Hercules, CA, USA).

### 2.8. Biochemical Indexes for Physiological Function of Visceral Organs

Four-week-old mice were divided into six groups. Groups 1–5 received oral administration of NC (300 μL), NP-Empty (without OmpA), OmpA (4 μg/g), NP-OmpA (4 μg/g), and NP-OmpA (8 μg/g), respectively. Group 6 received intraperitoneal injections of NP-OmpA (4 μg/g). After continuous oral administration for 7 days, mice serum and livers were taken. The liver tissues were centrifuged (900× *g*, 4 °C, 10 min) after homogenizing in ice-cold PBS, and the supernatants were assayed for alanine aminotransferase (ALT), aspartate transaminase (AST), catalase (CAT), glutathione (GSH), malondialdehyde (MDA), and superoxide dismutase (SOD) using commercial kits. Serum uric acid (UA) and creatinine (Cr) were measured according to the kit instructions (Jiancheng Institute of Biotechnology, Nanjing, China). Briefly, the samples were treated with different reagents, reacted under the specified conditions, 200 μL of the reaction solution was transferred to the wells, and the absorbance values at different wavelengths were determined with a microplate reader. The content or activity of each factor was calculated by the specific calculation formula of each factor [21].

### 2.9. Determination of Expression of Immune-Related Genes and Inflammation-Related Genes by qRT-PCR

mRNA was isolated from the spleen, liver, and kidney tissues using an RNA isolation kit (TAKARA, Tokyo, Japan) following the manufacturer’s instructions, as described previously [21]. Briefly, the mRNA was reverse-transcribed to cDNA using a PrimeScript RT Master Mix kit (TAKARA, Tokyo, Japan), and cDNAs were amplified using the primers shown in Table 1. The qRT-PCR was performed using an Applied Biosystems StepOnePlus™ Real-Time PCR System (ABI Applied Biosystems, Waltham, MI, USA) with a SYBR^®^ Green Permix Pro Taq HS qPCR kit (TAKARA, Tokyo, Japan). The relative quantitative method was used to obtain the gene expression of each factor. Briefly, ΔCt (Cycle threshold change) was obtained by comparing the difference between the Ct value of factor gene and an internal control gene (GAPDH), and then ΔΔCt was obtained by comparing the difference between the ΔCt value of experimental and control group. The mRNA expression was subsequently analyzed by the 2^−(ΔΔCt)^ formula.

### 2.10. Histopathological Morphology of Injury to Visceral Organs

The preparation of pathological sections of mice liver and kidney involved dehydration, transparency, sectioning, and hematoxylin and eosin (H&E) staining [21]. Briefly, the liver and kidney tissues were dehydrated using an alcohol gradient for 1 h and then placed in an alcohol:xylene mixture (1:1, *v/v*) for 30 min, xylene for 8 min, a xylene:paraffin solution (1:1, *v/v*) for 30 min, and paraffin for 1 h. Slices with a thickness of about 5 μm were cut, dried, H&E stained, observed under a microscope, and photographed (Leica, Wetzlar, Germany).

### 2.11. Statistical Analysis

All the experimental data were expressed as mean ± SD. The significant difference from the respective control in all experiments was assessed by one-way analysis of variance (ANOVA) using SPSS (IBM Corporation, Chicago, IL, USA). Values of *p* < 0.05 were considered statistically significant [21].

## 3. Results

### 3.1. Expression and Purification of Recombinant OmpA

**Figure 1:**
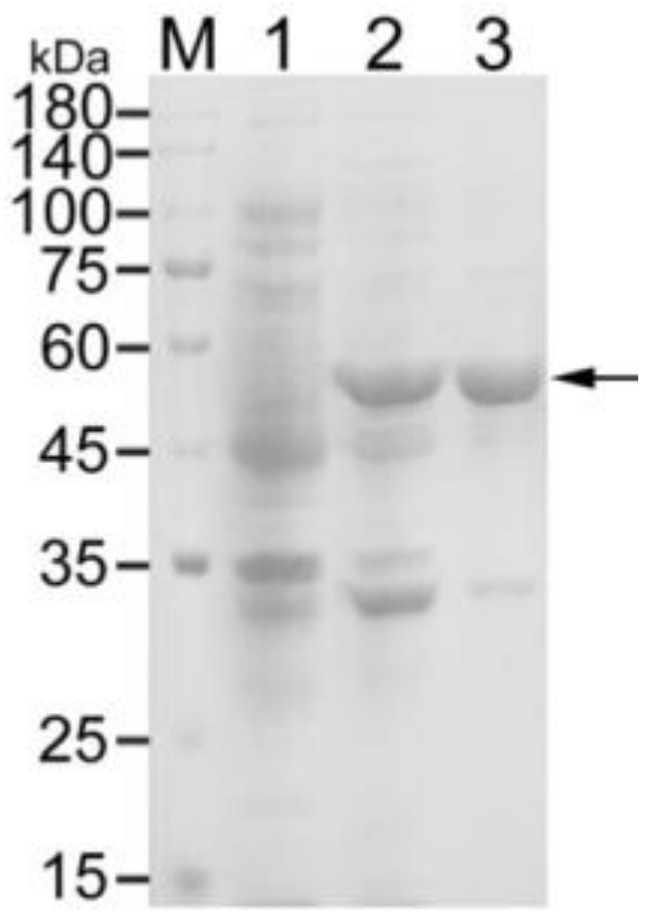
Purification of outer membrane protein A (OmpA). M, protein marker; 1, non-induced strain; 2, Isopropyl-β-d-thiogalactoside (IPTG)-induced strain; 3, purified OmpA.

Recombinant OmpA was obtained using Ni-NTA superflow resin. It had a molecular weight of about 60 kDa, comprised of the 39 kDa OmpA and the 20 kDa fusion protein (Figure 1).

### 3.2. Optimal Preparation Conditions for NP-OmpA

**Figure 2:**
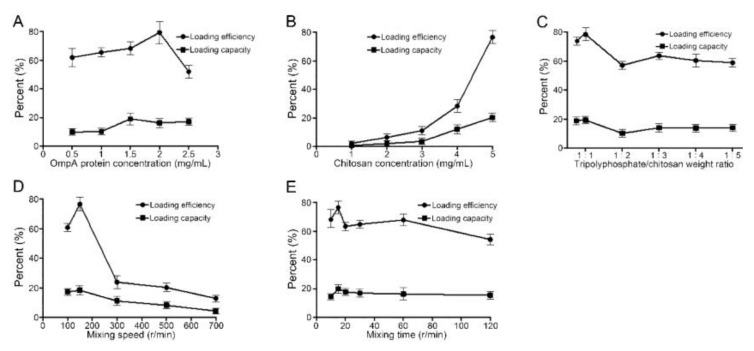
Effects of different factors on the preparation of NP-OmpA nanoparticles. (**A**) the optimal concentration of OmpA was 2 mg/mL; (**B**) the optimal concentration of chitosan (CS) was 5 mg/mL; (**C**) the optimal ratio of tripolyphosphate (TPP)/CS (*w/w*) was 1:1; (**D**) the optimal magnetic mixing speed was 150 r/min; (**E**) the optimal mixing time was 15 min.

The optimal preparation conditions for NP-OmpA were investigated by assessing the LE, LC, particle size, and morphology of the nanoparticles. The optimal concentration of OmpA was 2 mg/mL (Figure 2A). As the LE and LC increased when the CS concentration rose from 1 mg/mL to 5 mg/mL, the optimal CS concentration was determined as 5 mg/mL (Figure 2B), where LE and LC were 76.48% and 20.31%, respectively. The optimal ratio of TPP/CS (*w/w*) was 1:1, with a LE of 78.37% and LC of 19.31% (Figure 2C). The optimal magnetic mixing speed was 150 r/min, with LE of 76.59% and LC of 18.31% (Figure 2D). The optimal mixing time was 15 min, with LE of 76.42% and LC of 19.86% (Figure 2E).

### 3.3. Preparation of OmpA Nanoparticles

**Figure 3:**
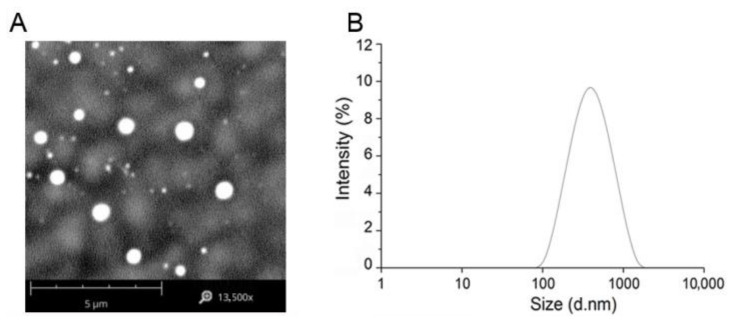
Morphology and particle size distribution of NP-OmpA. (**A**), scanning electron micrograph of NP-OmpA; (**B**), particle size distribution of NP-OmpA. The surface morphology and size distribution of NP-OmpA were uniform and spherical, and the particle size was about 700 nm.

NP-OmpA nanoparticles were prepared according to the optimal preparation condition. The OmpA nanoparticles (NP-OmpA) were 700.8 ± 14.6 nm in size, with uniformed spherical shape (Figure 3), and the zeta potential was 33.06 ± 1.15 MV, which showed that the NP-OmpA were stable. The LE was 79.27%, and the LC was 20.31%.

**Figure 4:**
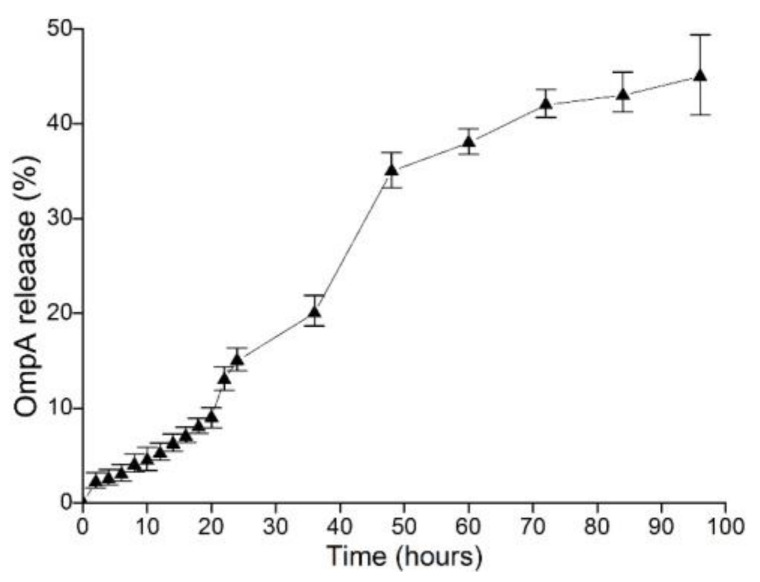
Characteristics of OmpA release from NP-OmpA in PBS (pH 1.2). The major release occurred from 0 to 48 h, and the release rate was <50%.

The gastric environment was simulated using PBS (pH 1.2) to assess the OmpA release from the NP-OmpA. The release was fast in the first 48 h, and slow down in the subsequent 48–96 h, reaching a final release rate of 43% at 96 h (Figure 4).

### 3.4. Effect of NP-OmpA on Mouse Liver and Kidney Functions

**Figure 5:**
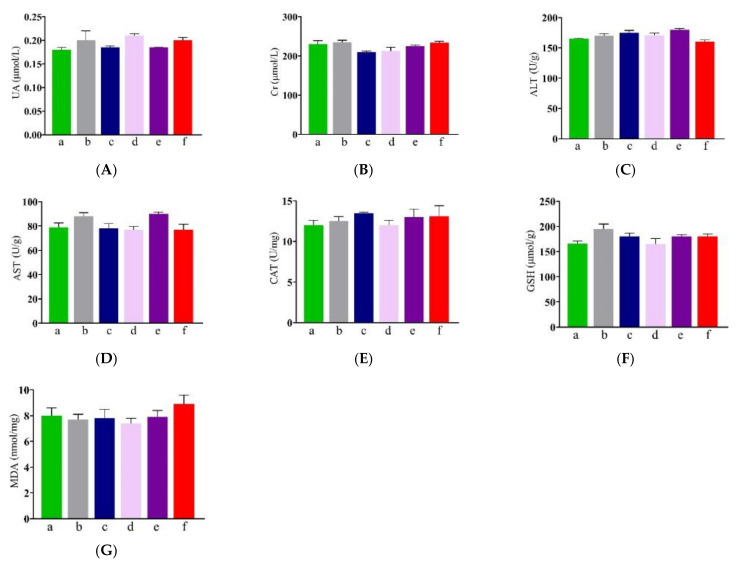
Effects of NP-OmpA immunization on liver and kidney functions in mice. (**A**,**B**) Serum UA and Cr were measured to investigate kidney function. (**C**,**D**) Liver ALT and AST were measured to investigate liver function. (**E**,**F**) Liver CAT and GSH were measured to determine the liver antioxidant index. (**G**) MDA was measured to determine the liver membrane lipid peroxidation index. Results of oral administration of normal saline (NC) (a), NP-Empty (b), 4 μg/g OmpA (c), 4 μg/g NP-OmpA (d), 8 μg/g NP-OmpA (e), and intraperitoneal injection of 4 μg/g NP-OmpA (f) are shown. There was no significant difference (*p* > 0.05) in uric acid (UA), creatinine (Cr), aminotransferase (ALT), aspartate transaminase (AST), catalase (CAT), glutathione GSH, and malondialdehyde (MDA) between the NP-OmpA group and the other groups.

NP-OmpA safety was assessed by examining the functional and antioxidant indexes of the kidneys and livers of treated mice. To investigate the kidney function index, serum UA and Cr were measured, and the results showed that there was no significant difference (*p* > 0.05) between mice immunized with NP-OmpA and OmpA, NC, and NP-Empty (Figure 5A,B). To investigate the liver function index, ALT and AST were measured, and no significant differences (*p* > 0.05) were found between NP-OmpA treated and control groups (Figure 5C,D). The liver antioxidant index (CAT and GSH) showed no significant differences (*p* > 0.05) among groups (Figure 5E,F). The liver membrane lipid peroxidation index, determined using MDA measurements, showed no significant differences (*p* > 0.05) among groups (Figure 5G).

### 3.5. Histopathological Observations of Tissues from Mice Immunized with NP-OmpA

**Figure 6:**
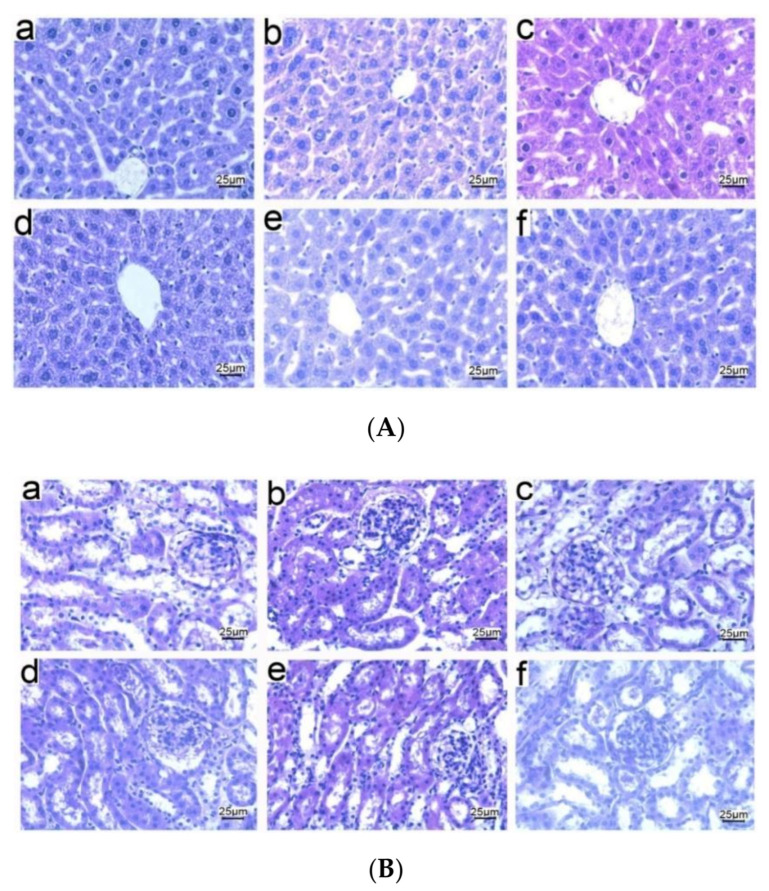
Histopathology of livers and kidneys of immunized mice. Liver (**A**) and kidney (**B**) sections are shown with hematoxylin and eosin (H&E) staining at ×400 magnification. Results of oral administration of NC (a), NP-Empty (b), 4 μg/g OmpA (c), 4 μg/g NP-OmpA (d), 8 μg/g NP-OmpA (e), and intraperitoneal injection of 4 μg/g NP-OmpA (f) are shown. Liver sections (**A**) show that every group had obvious hepatic sinusoids and cells with regular cell morphology, uniform cytoplasm, and clear nuclei. Kidney sections (**B**) show that every group had normal glomerular morphology, renal tubules arranged in order, and no obvious congestion or edema in the renal interstitium. The results showed that NP-OmpA had no toxic effect on mice livers and kidneys.

In order to assess the effect of NP-OmpA on the structure of the liver and kidneys, mice were immunized with NP-OmpA, and the tissue sections were prepared. The liver sections showed that NP-OmpA and control groups had obvious hepatic sinusoids and regular cell morphology, uniform cytoplasm, and clear nuclei (Figure 6A). The kidney sections showed that mice immunized with NP-OmpA and with control agents had normal glomerular morphology, renal tubules arranged in order, and no obvious congestion or edema in the renal interstitium (Figure 6B). The histological sections showed that the liver and kidney of mice immunized with NP-OmpA were intact, and NP-OmpA did not damage the organs of mice.

### 3.6. Immuno-Stimulating Activity of NP-OmpA

**Figure 7:**
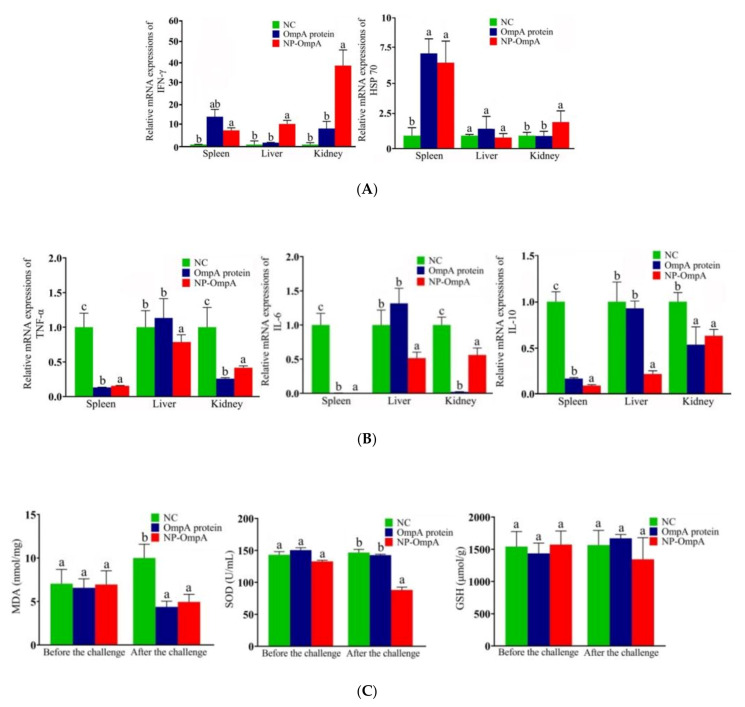
Analysis of immune-related factors before and after pathogenic *E. coli* challenge in mice. Labels a–c indicate statistically different groups (*p* < 0.05). (**A**) Effect of NP-OmpA on mRNA expression of immune-related factors before the challenge. Compared to NC and OmpA, NP-OmpA activated higher expression levels of IFN-γ and HSP70 in the spleen, liver, and kidney. (**B**) Effect of NP-OmpA on mRNA expression of inflammation-related factors after challenge. Compared to NC, NP-OmpA and OmpA resulted in decreased expression levels (*p* < 0.05) of TNF-a, IL-6, and IL-10 in the spleen, liver, and kidney, especially in the spleen. (**C**) Levels of antioxidant-related factors before and after *E. coli* challenge. After the challenge, animals immunized with NP-OmpA and OmpA had lower MDA and SOD liver levels than those that received NC, and the level was lower in those immunized with NP-OmpA compared to OmpA.

After seven days of the fourth post-vaccination, the mRNA expression of immune-related genes of IFN-γ and HSP70 were evaluated by qRT-PCR to analyze NP-OmpA immune activation in mice. Compared with the NC and OmpA control groups, the NP-OmpA group (orally administered 6 μg/g) activated higher expression levels of IFN-γ and HSP70 in the spleen, liver, and kidney (Figure 7A).

In order to assess NP-OmpA’s protective effect, the mRNA expression of inflammation-related genes of TNF-a, IL-6, and IL-10 in the spleen, liver, and kidneys was evaluated after the *E. coli* challenge to mice. The results showed that the expression levels of TNF-a, IL-6, and IL-10 were decreased (*p* < 0.05) in the spleen, liver, and kidney compared with the control group, especially in the spleen (Figure 7B).

Antioxidant-related factors were evaluated in the liver after two days of mice challenged with *E. coli*. Animals immunized with NP-OmpA and OmpA had lower MDA and SOD liver levels than those that received NC. The level was lower in those immunized with NP-OmpA compared to those that received OmpA (Figure 7C).

The phagocytosis of WBC was evaluated using *S. aureus* as it possesses a round shape for convenient counting. Regarding the thymus index, spleen index, and phagocytic percentage (*PP*), the measurements in the NP-OmpA group were higher than those of the other groups, and the thymus index reached significance (*p* < 0.05). The spleen index and *PP* of the NP-OmpA and OmpA groups were significant (*p* < 0.05) compared to the other groups (Table 2).

**Figure 8:**
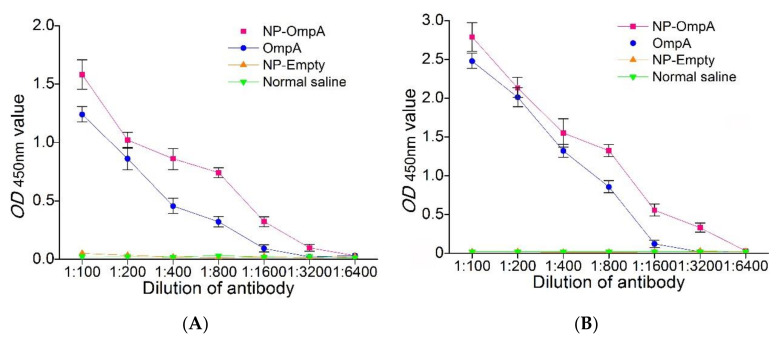
ELISA results showing the interaction between antibodies and *Escherichia coli* (**A**) and antibody titer (**B**). Panel A shows that antibodies from mice immunized with NP-OmpA interacted with *E. coli* when the dilution reached 1:1600, and the binding ability of antibodies from mice immunized with NP-OmpA to *E. coli* was greater than that of antibody from other groups in vitro. Panel B shows that antibodies from mice immunized NP-OmpA bound to OmpA at a dilution of 1:3200 were at a higher titer than other groups.

ELISA results showed that antibodies from mice immunized with NP-OmpA interacted with *E. coli* when the dilution reached 1:1600, which was higher than OmpA (1:800), as well as NP-Empty and NC groups (Figure 8A). Mice immunized with NP-OmpA were found to have antibodies that bound to activated OmpA at a dilution of 1:3200, which was a higher titer than mice immunized with OmpA, NP-Empty, and NC (Figure 8B). The results indicated that NP-OmpA antiserum had a higher titer, and it was able to recognize *E. coli*.

### 3.7. Immunoprotective Effect of NP-OmpA

**Figure 9:**
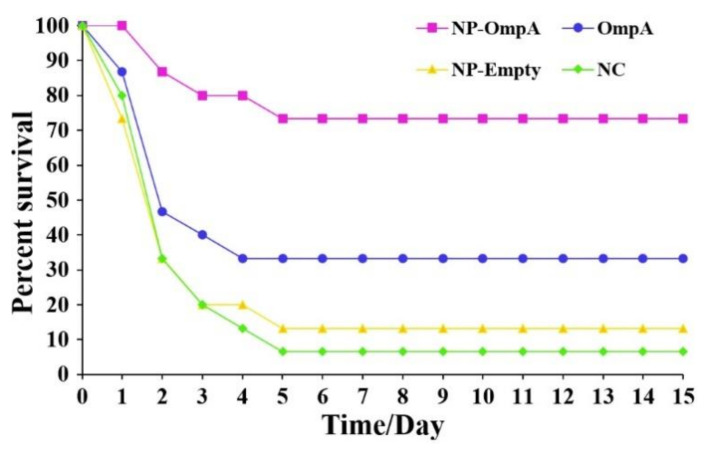
After challenge with *E. coli*.

Mice were orally immunized with NP-OmpA, OmpA, NP-Empty, and NC, respectively. The immunoprotective effect was evaluated by challenge with *E. coli*. Mice in each group developed severe toxic symptoms, including fluffy folds, sluggish activity, listlessness, and lethargy. Furthermore, the majority of the mice in the control groups died within three days, and the other mice survived to day five and later with gradual recovery (Figure 9). The immune protection rate of NP-OmpA immunization (71.43%) was significantly higher (*p* < 0.05) than that of OmpA (28.57%), NP-Empty (7.14%), and NC (Table 3).

### 3.8. Liver and Kidney Histopathology of Mice Challenged with E. coli

**Figure 10:**
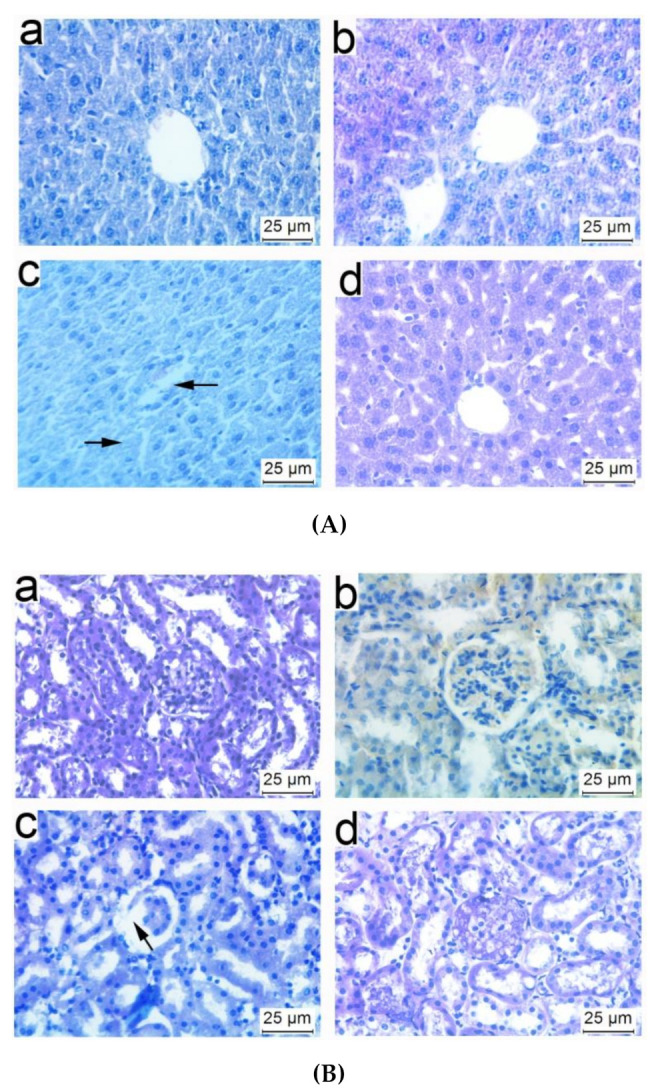
Histopathology of livers and kidneys of mice after immunization and *E. coli* challenge. Liver (**A**) and kidney (**B**) sections are shown with H&E staining at ×400 magnification. Mice were immunized with NP-OmpA (a), OmpA (b), or NC (c) and then challenged with *E. coli*, or immunized with NC and challenged with NC (d). After *E. coli* challenge, inflammatory cell infiltration in the central vein of liver tissue, nuclear apoptosis of liver cells, irregular cell morphology, uneven cytoplasm, and unclear hepatic sinuses were observed (Ac); the incomplete structure of kidney tissue, irregular glomerular and atrophy, and loose cells were observed (Bc). Thus, immunization with NP-OmpA can reduce the injury to the liver and kidney caused by *E. coli*.

Liver and kidney sections were prepared after two days after the mice were challenged with *E. coli* to observe any injury. Compared to the livers of mice immunized with NP-OmpA (orally administered 6 μg/g), OmpA, and negative control (without *E. coli* challenge), livers of mice that received NC appeared to have inflammatory cell infiltration in the central vein, nuclear apoptosis, and unclear hepatic sinuses after challenge (Figure 10A), and the kidneys of mice received NC appeared to have glomerular atrophy (Figure 10B). Furthermore, the livers and kidneys of mice immunized with NP-OmpA appeared to have less injury than those of mice immunized with OmpA.

## 4. Discussion

Nanomaterials exhibit specific properties or functions, which has attracted extensive attention in biomedicine studies. Active compounds can be coated with specific materials to prepare nanoparticles, and these coating materials can help to promote and maintain the biological activity of the encapsulated compounds, facilitate sustained release, change the route of administration, improve drug utilization, reduce adverse reactions, and they can be degraded and absorbed by the host [22,23]. When applied to oral vaccination, nanoparticles can protect drugs from degradation induced by gastrointestinal enzymes and low pH values, improve the bioavailability of drugs, and enhance drug functions [24,25]. CS is a natural amino polysaccharide resulting from the deacetylation of chitin and has high biodegradability and biocompatibility, making it useful for broad purposes in medicine, food, textiles [26,27]. Khouloud et al. used CS to encapsulate whey protein and prepare whey-CS nanoparticles, and this formulation improved the stability of whey protein [28]. CS was used to encapsulate immobilizing glucoamylase nanoparticles, which retained 80% activity after four months [29]. *Edwardsiella tarda* OmpA was encapsulated with CS, and after treatment, the post-challenge survival proportion (PCSP) was 73.3%, and the nanoparticles could enhance immunological function [30]. In this study, OmpA was purified by Ni-NTA slurry and encapsulated with CS to prepare NP-OmpA nanoparticles with diameters of about 700 nm. The optimal preparation conditions for NP-OmpA included an OmpA concentration of 2 mg/mL, a CS concentration of 5 mg/mL, a ratio of TPP/CS (*w/w*) of 1:1, a magnetic mixing speed of 150 r/min, and a mixing time of 15 min. In acidic solution (pH 1.2), the release rate of the NP-OmpA (0~96 h) was less than 50%, indicating that NP-OmpA was stable in gastric fluid. Thus, NP-OmpA has value in oral delivery applications.

Internalized NP-OmpA may affect the host’s health; therefore, it is necessary to evaluate visceral organ function and injury. UA and Cr levels were mainly used to determine kidney function indexes, and ALT, AST, CAT, GSH, and MDA levels were used to evaluate liver function. Using the levels of MPO, SOD, MDA, GSH-Px, GSH, and MDA, and immunohistochemical and immunofluorescence analyses, Lu et al. evaluated the protective effects of dexmedetomidine on lipopolysaccharide-induced acute lung injury [31], and Ezz-Eldin et al. assessed the possible protective effect of carvacrol against bronchial asthma induced experimentally in rats [32]. Alhusaini et al. found that the intake of N-acetylcysteine (NAC) and thymoquinone (THQ) could protect against the nephrotoxicity induced by sodium fluoride (NAF) by analyzing SOD, GSH, UA, and Cr [33]. Our results showed that there were no significant differences in serum UA and Cr, in liver ALT, AST, CAT, GSH, MDA, or in the liver and kidney sections between mice immunized with NP-OmpA and the control groups. These results suggested that NP-OmpA had no toxic effect on mice livers and kidneys.

Animals can be immunized with protegrin to enhance immune function and to increase resistance to pathogen infection [34,35]. In our safety evaluation, NP-OmpA was divided into concentration gradient (4 μg/g and 8 μg/g) and subjected to different administration methods (i.p. and oral); we collected comprehensive biological indexes and could confirm that NP-OmpA was safe for mice in different regimes, in order to optimize the study design and to reduce unnecessary mice investigation [30], we set the NP-OmpA concentration at 6 μg/g for oral administration in the subsequent study. This study found that NP-OmpA could significantly increase the immune-related gene expression of IFN-γ and HSP70 and the phagocytic activity to *S. aureus* of WBC in mice, which showed that the non-specific immune function was enhanced [36]. *S. aureus* possesses a round shape for convenient counting and has been widely used in the analysis of leukocyte phagocytosis, is also a major etiologic agent of cow mastitis [1], and immunization with NP-OmpA may boost resistance to *S. aureus* infection. Moreover, we found that antiserum from animals immunized with NP-OmpA recognized with *E. coli*, which suggested that anti-NP-OmpA antibodies and *E. coli* formed antigen-antibody complexes and likely enhanced antigen presentation [15]. Antibodies from mice immunized with NP-OmpA also bound to OmpA at a higher titer (3200) than those from mice immunized with OmpA. The results suggested that NP-OmpA could enhance the immune activity of mice.

The immune protection function of the protein against bacterial infection can be evaluated by immunizing mice with the protein and challenging the mice with the pathogenic bacteria, and then analyzing the death rate [16], visceral organ injury [37], and expression of inflammation-related genes and antioxidant factors [38,39]. Our study showed that the immune protection effect of NP-OmpA was 71.43% (*p* < 0.05), which was higher than that of OmpA (28.57%), NP-Empty (7.14%), and the NC group. Purified OmpA possessing a 20 kDa protein tag, which could enhance the expression of the target protein and has been widely used. In our previous study, we compared the immune activity of this tag with normal saline and did not find a difference [16]. Thus, in the current study, we did not use the nanoparticle-encapsulated 20 kDa protein tag in the control group [30]. Different timing of challenge may have led to different outcomes; therefore, we set the timing of blood and tissue sample collection at day seven after the last booster (the fourth immunization) in both NP-OmpA and control groups; therefore, the variation caused by challenge timing could be minimized. After mice were challenged with *E. coli*, immunization with NP-OmpA was shown to decrease the expressions of the inflammation-related genes TNF-a, IL-6, and IL-10, and the expression of antioxidant factors MDA and SOD was also decreased, which indicated that immunization with NP-OmpA could reduce the inflammatory reaction caused by *E. coli*. Moreover, an examination of histopathological sections showed that immunization with NP-OmpA could reduce injury to mouse livers and kidneys caused by *E. coli*. Thus, NP-OmpA was higher than OmpA in responses to critical immune indexes, including immune protection effect, inhibition of the expression of inflammation-related genes, thymus index, and antibody titer.

## 5. Conclusions

Novel nanoparticles (NP-OmpA) were encapsulated, and the preparation method was optimized. The analysis of antioxidant factors and histopathological observation confirmed that the NP-OmpA was safe for mice, and the immune protection rate was 71.43% (*p* < 0.05). Immunization with NP-OmpA could enhance the expression of immune factors and leukocyte phagocytosis of *S. aureus.* A high antiserum titer was obtained from mice immunized with NP-OmpA, and antibodies recognized *E. coli*. NP-OmpA vaccination could down-regulate the expression of inflammation-related genes and antioxidant factors and reduce visceral organ injury induced by *E. coli*. This study contributes to the development of an orally delivered nanoparticle that could be used to boost resistance to infection with the etiologic agents of cow mastitis.

## Figures and Tables

**Table 1:** Primers used for qRT-PCR.

Gene	Forward Primer (5′-3′)	Reverse Primer (5′-3′)
IFN-γ	TCAAGTGGCATAGATGTGGAAGAA	TGGCTCTGCAGGATTTTCATG
HSP70	GAAGGTGCTGGACAAGTGC	GCCAGCAGAGGCCTCTAATC
TNF-a	TATGGCTCAGGGTCCAACTC	GCTCCAGTGAATTCGGAAAG
IL-6	GACAAAGCCAGAGTCCTTCAGAGAGATACAG	TTGGATGGTCTTGGTCCTTAGCCAC
IL-10	AACATACTGCTAACCGACTC	ATGCTCCTTGATTTCTGG
GAPDH	ACAGTCCATGCCATCACTGCC	GCCTGCTTCACCACCTTCTTG

**Table 2:** WBC, organ index, and leukocyte phagocytosis of *S. aureus* values.

Group	Compound	WBC No. (×10^9^/L)	Thymus Index	Spleen Index	Phagocytic Percentage (*PP* %)	Phagocytic Index (*PI %*)
1	NP-OmpA	6.83 ± 0.97 ^a^	2.67 ± 0.12 ^b^	4.67 ± 0.38 ^b^	5.76 ± 1.10 ^b^	3.56 ± 0.62 ^a^
2	Control 1 (OmpA)	6.93 ± 0.89 ^a^	2.25 ± 0.10 ^a^	4.01 ± 0.41 ^ab^	4.85 ± 0.33 ^ab^	3.67 ± 0.42 ^a^
3	Control 2 (NP-Empty)	6.73 ± 0.48 ^a^	2.08 ± 0.07 ^a^	3.48 ± 0.43 ^a^	4.68 ± 1.02 ^a^	3.08 ± 0.43 ^a^
4	Control 3 (Normal saline)	6.68 ± 0.67 ^a^	2.03 ± 0.11 ^a^	3.31 ± 0.25 ^a^	4.21 ± 0.98 ^a^	3.16 ± 0.58 ^a^

Labels a,b indicates statistically different groups (*p* < 0.05). Mice immunized with NP-OmpA recorded higher thymus index, spleen index, and *PP* values than other groups, indicating that NP-OmpA had immuno-stimulating activity.

**Table 3:** Active immune protection observed in mice.

Group	Compound	Nos	Survival No.	Death No.	ADR, %	RPS, %
1	NP-OmpA	15	11	4	26.67	71.43 **
2	Control 1 (OmpA)	15	5	10	66.67	28.57
3	Control 2 (NP-Empty)	15	2	13	86.67	7.14
4	Control 3 (Normal saline)	15	1	14	93.33	—

Notes: ADR, accumulated death rate. RPS, immune protection rate. RPS (%) = 1 − (% vaccinated mortality/% non-vaccinated mortality) × 100. ** *p* < 0.01 (compared with control 3). The highest RPS was in the NP-OmpA-immunized group (71.43%), followed by the OmpA-immunized group (28.57%), while the NP-Empty-immunized group (7.14%) had the lowest RPS. There was a significant difference (*p* < 0.05) between groups immunized with NP-OmpA and with OmpA, NP-Empty, and NC.

## Data Availability

The data presented in this study are available within the article.
